# The Importance of Reading the Skin: Cutaneous Metastases of Pancreatic Cancer, a Systematic Review

**DOI:** 10.3390/jcm13010104

**Published:** 2023-12-24

**Authors:** Fortunato Cassalia, Anna Bolzon, Monica Ponzano, Laura Ventura, Andrea Danese, Paolo Del Fiore, Anna Belloni Fortina, Elio Jovine, Giampaolo Perri, Umberto Cillo, Giovanni Marchegiani

**Affiliations:** 1 Dermatology Unit, Department of Medicine [DIMED], University of Padua, 35121 Padua, Italy; fortunato1287@gmail.com (F.C.); annabolzon44@gmail.com (A.B.); monicaponzano@live.com (M.P.); anna.bellonifortina@gmail.com (A.B.F.); 2 Department of Statistics, University of Padua, 35121 Padova, Italy; ventura@stat.unipd.it; 3 Section of Dermatology and Venereology, Department of Medicine, University of Verona, 37129 Verona, Italy; adanese4@gmail.com; 4 Soft-Tissue, Peritoneum and Melanoma Surgical Oncology Unit, Veneto Institute of Oncology IOV-IRCCS, 35128 Padua, Italy; paolo.delfiore@iov.veneto.it; 5 Department of Woman’s and Children’s Health, University of Padua, 35121 Padua, Italy; 6 Department of General Surgery, IRCCS, Azienda Ospedaliero-Universitaria di Bologna, Maggiore Hospital, 40133 Bologna, Italy; elio.jovine@ausl.bologna.it; 7 Hepatopancreatobiliary and Liver Surgery, Department of Surgery, Oncology and Gastroenterology DiSCOG, University of Padua, 35121 Padua, Italy; giampaolo.perri@unipd.it (G.P.); cillo@unipd.it (U.C.)

**Keywords:** cutaneous metastasis, skin metastasis, pancreatic cancer, pancreatic skin metastasis, pancreas, skin cancer, pancreatic cancer early detection, skin metastasis from pancreatic cancer

## Abstract

Background: Pancreatic cancer is notorious for its aggressive nature and low survival rate, with less than 10% of patients surviving beyond five years. Early detection is difficult, but skin metastases can be a rare but significant indicator. This systematic review focuses on the epidemiology, clinical features, and histology of skin metastases from pancreatic cancer to determine their importance in early diagnosis and overall management of the disease. Materials and methods: Following PRISMA guidelines, we conducted an exhaustive search of MEDLINE/PubMed, EMBASE, and SCOPUS databases up to June 2023, using specific keywords. Four independent investigators screened the studies using predefined criteria, and two investigators checked the accuracy and consistency of the data extraction. We assessed the quality of the trials using adapted criteria from the Joanna Briggs Institute. A narrative synthesis rather than a meta-analysis was chosen because of the different study designs. Results: The final analysis included 57 patients with skin metastases from pancreatic cancer. Cutaneous metastases, although rare, presented with approximately equal gender distribution and a mean age of 63.4 years. Predominantly non-umbilical (77%), these metastases showed clinical diversity, ranging from asymptomatic nodules to painful or ulcerated lesions. Notably, skin metastases often preceded the diagnosis of primary pancreatic cancer (58%). Primary tumor characteristics revealed different localizations, with adenocarcinoma being the most prevalent histological type (77%). A significant association (*p* = 0.008) was observed between pancreatic tumor location and the timing of presentation of skin metastases. Tumors located in the body and tail of the pancreas were more likely to manifest skin metastases as an initial clinical manifestation (62.2%) than those in the head of the pancreas (20.8%). Conclusions: In conclusion, although skin metastases are rare, they are important indicators of pancreatic cancer, highlighting the need for multidisciplinary healthcare collaboration and thorough skin examination. Recognizing them could lead to earlier diagnosis, which is crucial in a cancer with limited treatment options.

## 1. Introduction

Pancreatic cancer is a highly aggressive neoplasm with a poor prognosis due to its aggressive nature and the difficulty of early diagnosis. It is currently the seventh leading cause of cancer death worldwide, with a five-year survival rate of less than 10% [1,2]. Pancreatic cancer is characterized by an aggressive nature, early metastatic potential, and resistance to treatment [3]. Histotypes of pancreatic cancer include pancreatic ductal adenocarcinoma, which is the most common subtype and arises from ductal cells [4]. Less common are neuroendocrine tumors, which arise from hormone-producing cells and vary in malignancy [5]. Rare histotypes include acinar cell carcinoma, which histologically mimics pancreatic acinar cells; adenosquamous carcinoma, also rare, is characterized by glandular and squamous cells and is very aggressive; and anaplastic carcinoma of the pancreas, which is extremely rare and remarkably aggressive, with a poor prognosis [6,7]. While metastases typically involve the liver, peritoneum, and lungs, skin metastases (CM) are a rare manifestation of advanced pancreatic cancer, accounting for only 1–3% of metastases from pancreatic cancer. According to the literature, the most common site for cutaneous metastases is the navel. These metastases, which often appear as firm, nodular lesions, are called “Sister Mary Joseph nodules”. Other reported sites for cutaneous pancreatic metastases are the head and neck, chest, abdomen, buttocks, scrotum, and labia [8,9,10,11]. Skin metastases from pancreatic cancer may present in various forms, such as nodules or subcutaneous masses and, less frequently, as plaques, erythematous patches, or skin thickening. These lesions may be solitary or multiple, painful, or asymptomatic, and may be accompanied by systemic symptoms such as abdominal pain, weight loss, jaundice, and anorexia [12]. In rare cases, skin metastases from pancreatic cancer may be the first manifestation of the disease, preceding the diagnosis of the primary [13], while in other cases they may be the first sign of recurrence. Differential diagnoses include malignant conditions such as primary cutaneous neoplasms or skin metastases from other primary neoplasms, as well as benign conditions such as infections, autoimmune disorders such as granulomatous skin diseases, and benign causes such as navel hernias or, rarely, endometriosis, pyoderma gangrenosum, or similar. Sometimes, they may resemble post-inflammatory or post-surgical processes, such as keloids [14]. The diagnosis of skin metastases from pancreatic cancer is typically made by biopsy of the skin lesion [15]. To assess the extent of metastatic disease and identify other sites of involvement, imaging studies such as CT, PET-CT, endoscopic ultrasound, or MRCP (magnetic resonance cholangiopancreatography) are indicated [16,17,18]. The management of skin metastases from pancreatic cancer focuses mainly on palliative care and the patient’s quality of life. Surgical excision of a skin metastasis is only considered in highly selected cases, with the goal of radical resection after removal of the primary tumor, and the skin is the only site of distant lesions. However, the efficacy of these treatments is limited, and the response rate is low [19,20,21]. The aim of this systematic review is to analyze the literature to further our knowledge of the clinical features of skin metastases from pancreatic cancer. It aims to provide guidance to physicians facing rare skin metastases from pancreatic cancer, helping them to choose the most appropriate diagnostic and therapeutic approach in the shortest possible time. Given the high aggressiveness of this disease, it is crucial to save time in the patient management process. Furthermore, this review seeks to confirm the most common site for skin metastases from pancreatic cancer and to explore possible correlations between skin metastases and the location of the primary tumor.

## 2. Materials and Methods

### 2.1. Study Design

**Figure 1:**
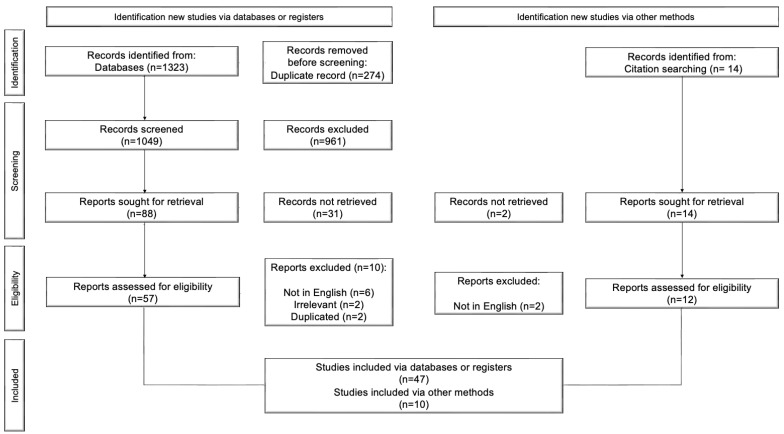
PRISMA flow chart.

This systematic review follows the Preferred Reporting Items for Systematic Reviews and Meta-Analyses (PRISMA) guidelines (Figure 1). 

### 2.2. Search Strategy

A comprehensive literature search was conducted in three major databases, namely MEDLINE/PubMed, EMBASE, and SCOPUS, without any language restrictions. The search strategy was executed using the following search string: “cutaneous metastasis from pancreatic cancer OR skin metastases from pancreatic cancer OR cutaneous metastasis from pancreatic neoplasms OR skin metastases from pancreatic neoplasms”. The search was performed up until June 2023. We included only case reports and small case series published in English that accurately described the characteristics of pancreatic cancer skin metastases. To maintain consistency, the search strategy was adapted to each electronic database. After retrieving the search results, we combined the lists from each source and removed any duplicate records. Four independent investigators (FC, AB, MP, and AD) screened the titles and abstracts of the remaining records to exclude those not relevant to the scope of this review. Subsequently, the full-text articles of potentially eligible records were assessed to determine their inclusion based on the predefined criteria. Furthermore, the reference lists of included articles were manually searched to identify additional relevant studies. Any discrepancies between the investigators were resolved through consensus, involving the external opinion of a fifth investigator. Studies that did not involve human subjects were excluded. Language restrictions were not applied during the selection process.

### 2.3. Inclusion Criteria

The inclusion criteria for this systematic review were carefully constructed according to the PRISMA guidelines. Only case reports and small case series published in English that accurately described the characteristics of pancreatic cancer skin metastases were included. A key requirement was that each study was available in its entirety to allow us to examine its details. We also paid close attention to the relevance of each study, screening titles and content to ensure they were relevant to our research topic. This approach allowed us to create a focused and comprehensive collection of literature relevant to our study. The results of the search and screening are summarized in the PRISMA flowchart. The search strategy yielded 1323 unique citations, of which 274 were excluded as duplicates. The remaining 1049 articles were screened for eligibility using titles and abstracts, and 992 articles were excluded. Of the total number of reports assessed for eligibility (n = 57), 10 were excluded (6 because not in English, 2 because irrelevant, and 2 because duplicates). A total of 10 articles were added through the citation search. A total of 57 articles were included in the review.

### 2.4. Data Collection

Four investigators (AB, AD, FC, and MP) independently extracted relevant data from the included articles. For each study, we collected information on study features, patient characteristics, tumor details, and outcome measures. Another different investigator (PDF) cross-checked the extracted data to ensure accuracy and consistency. In cases of any discrepancies, a consensus was reached among all investigators.

### 2.5. Assessment of the Quality of Included Studies

The quality assessment of the included studies was conducted using eight predefined criteria, adapted from the Joanna Briggs Institute (JBI) critical appraisal tool [ref] to suit the context of this review. The criteria include (i) clear inclusion criteria for patients, (ii) valid methods for identifying the diagnosis, (iii) consecutive inclusion of patients in case series, (iv) clear reporting of demographics, and (v) clear reporting of clinical information. Three investigators (LV, UC, and GM) independently appraised the risk of bias in the included studies, and any discrepancies were resolved through consensus involving all authors.

### 2.6. Data Synthesis

The selection process will be presented in a flow chart. Pertinent data extracted from the included studies will be summarized in tables. Due to the inclusion of case reports and small case series, which may limit the feasibility of a meaningful meta-analysis, a narrative synthesis of the included studies will be conducted.

### 2.7. Statistical Analysis

The qualitative and dichotomized quantitative variables were expressed as counts and percentages in each category. Associations between categorical variables were analyzed in bivariate analysis with Pearson’s chi-square test with simulated *p*-value. Survival curves were estimated with the Kaplan–Meier method and were compared with the log-rank test. Statistical analysis was performed using R for MacOS (R statistical software version 4.1.0; R Core Team). The level of significance was set at *p* < 0.05.

## 3. Results

### 3.1. Study Selection and Cohort Characteristics

We conducted a systematic review following the PRISMA guidelines. Our search across MEDLINE/PubMed, EMBASE, and SCOPUS yielded a final cohort of 57 patients with cutaneous metastases from pancreatic cancer, as reported in various publications up until June 2023.

### 3.2. Epidemiology

**Figure 2:**
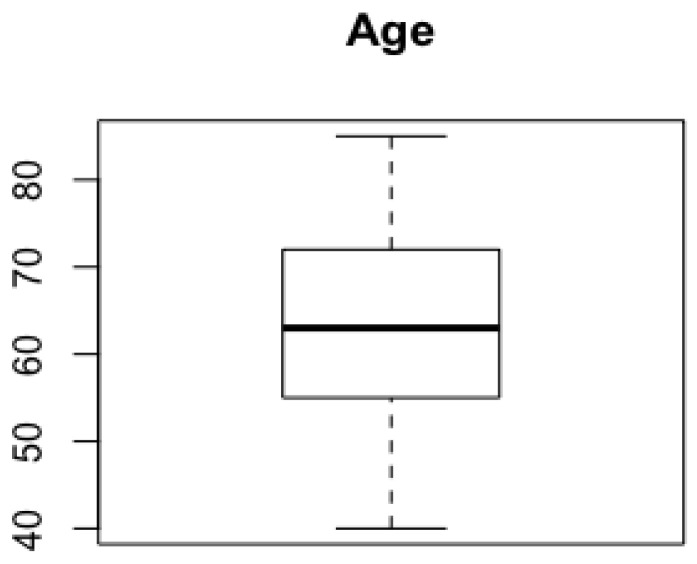
Age distribution.

Among these patients, 51% (n = 29) were females, and 49% (n = 28) were males, with a mean age of 63.4 years (SD = 12.0, Figure 2).

### 3.3. Characteristics of Cutaneous Metastases (Table 1)

Cutaneous metastases were predominantly non-umbilical (77% of cases, n = 44), with localized umbilical metastases being less common (23% of cases, n = 13). Among non-umbilical metastases, the most frequent sites were the head and neck (33%, n = 19), followed by the trunk (21%, n = 12), multiple districts (12%, n = 7), lower limbs (7%, n = 4), upper limbs (4%, n = 2), and genital skin (2%, n = 1). Clinically, most skin metastases presented as nodules/masses/plaques (89% of cases, n = 51), with swelling in 7% (n = 4) and ulceration in 4% (n = 2). Metastases were asymptomatic in 34% of cases (n = 21), associated with pain in 34% of cases (n = 14), and ulceration/bleeding in 19% of cases (n = 10). Most skin lesions were larger than 1 cm (78% of cases, n = 39), with 22% of cases (n = 11) having lesions smaller than 1 cm. Isolated metastases were more common (68% of cases, n = 39) than multiple metastases (32% of cases, n = 18).

**Table 1:** Cutaneous metastases characteristics in n = 57 patients.

Location	Count	Percentage
Umbilicus	13	23%
Other Sites	44	77%
Head And Neck	19	33%
Trunk	12	21%
Multiple Sites	7	12%
Lower Limbs	4	7%
Upper Limbs	2	4%
Genital Skin	1	2%
**Clinical Appearance**		
Nodule/Mass/Plaque	51	89%
Swelling	4	7%
Ulceration	2	4%
**Associated Symptoms**		
No Symptoms	21	34%
Pain	14	34%
Bleeding	10	19%
**Dimension**		
>1 cm	39	78%
<1 cm	11	22%
**Number**		
Isolated Metastases	39	68%
Multiple Metastases	18	32%
**Diagnosis**		
Before Pancreatic Cancer	33	58%
After Pancreatic Cancer	23	40%
Concomitant To Pancreatic Cancer	1	2%

### 3.4. Timing of Diagnosis 

In 58% of cases (n = 33), cutaneous metastases were detected before the diagnosis of pancreatic cancer, while in 40% of cases (n = 23), the patient had already been diagnosed with pancreatic cancer before cutaneous metastases were identified. Only one case (2%) had a synchronous diagnosis of pancreatic cancer and cutaneous metastases.

### 3.5. Pancreatic Cancer Characteristics (Table 2)

Primary pancreatic cancer was localized to the body or tail of the pancreas in 44% of cases (n = 25), whereas it was localized to the head in 42% of cases (n = 24). The primary tumor dimension was reported in 21 patients, with the mass being smaller than 4 cm in 52% of cases (n = 11) and larger than 4 cm in 48% of cases (n = 10). Histology indicated that most skin metastases were associated with pancreatic adenocarcinoma (77% of cases, n = 40). In 7% of cases (n = 4), the metastases were from pancreatic neuroendocrine cancer, in 4% (n = 2) from pancreatic sarcoma, and in 2% (n = 1) from pancreatic ductal mucinous adenocarcinoma. Detailed histology was not reported in 10% of cases (n = 5).

**Table 2:** Pancreatic cancer characteristics in n = 57 patients with cutaneous metastases.

Primary Site	Count	Percentage
Head	24	42%
Body and Tail	25	44%
**Dimension**		
<4 cm	11	52%
>4 cm	10	48%
**Histology of skin metastases**		
Adenocarcinoma	40	77%
Neuroendocrine cancer	4	7%
Sarcoma	2	4%
PDAC	1	2%
**Signs and symptoms**		
Gastrointestinal symptoms	23	44%
Jaundice	10	20%
Weight loss	10	20%
**Lymph node metastases**		
No	26	55%
Yes	21	45%
**Distant metastases**		
No	14	26%
Yes	39	74%
Liver	21	54%
Lungs	12	31%
Peritoneum	7	18%
Bones	5	13%
Portal vein	2	5%

### 3.6. Clinical Presentation 

At the time of diagnosis of skin metastases from pancreatic cancer, 44% of cases (n = 23) presented with gastrointestinal symptoms, while jaundice and weight loss were observed in 20% of cases (n = 10).

### 3.7. Metastasis and Treatment 

In terms of the staging of pancreatic cancer extension, 55% of cases (n = 26) did not have lymph node metastases, while 74% of cases (n = 39) had distant metastases. The most frequent distant metastases were hepatic metastases (43% of cases, n = 21), followed by lung metastases (32% of cases, n = 12). Peritoneal metastases were observed in seven cases, bone metastases in five cases, and portal vein metastases in two cases. Palliative therapy was administered in 23% of cases (n = 9), while a curative approach with chemotherapy (CT) or radiotherapy (RT) was carried out in 77% of patients (n = 31).

### 3.8. Statistical Analysis 

Statistical analysis included bivariate analysis with Pearson’s chi-square test, survival analysis with Kaplan–Meier curves, and comparison using the log-rank test, all performed using R for MacOS (version 4.1.0). No statistically significant correlations were found between the site of metastasis and the location of pancreatic cancer (head or body/tail) (*p* value = 0.7), nor between the site of pancreatic cancer and patient survival (*p* value = 0.4).

### 3.9. Association between Pancreatic Cancer Site and Cutaneous Metastasis as First Presentation 

**Figure 3:**
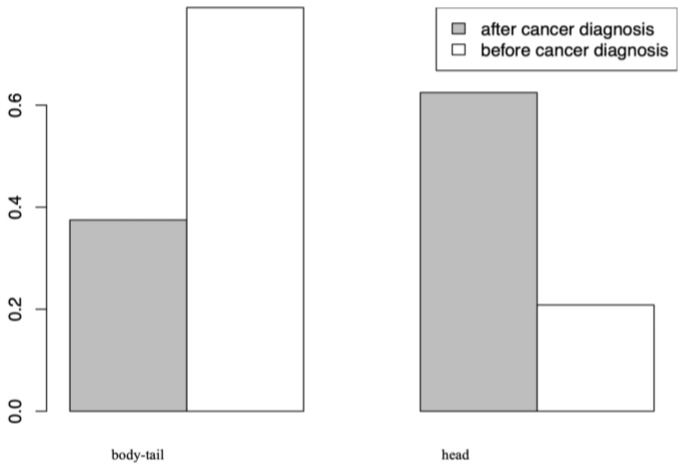
Timing of skin metastasis—Pancreatic site involved.

Our analysis revealed interesting insights into the relationship between pancreatic cancer sites and the occurrence of skin metastases as initial presentation. Using Pearson’s Chi-square test, we identified a statistically significant association between these variables (*p*-value = 0.008415). Patients with pancreatic tumors located in the body and tail of the pancreas were significantly more likely (62.2%) to have skin metastases as their first clinical manifestation than those with tumors in the head of the pancreas (20.8%) (Figure 3).

## 4. Discussion

### 4.1. Discussion 

Pancreatic cancer is one of the most aggressive malignancies, characterized by an insidious nature and a challenging early diagnosis [22]. This disease ranks as the seventh leading cause of cancer-related deaths worldwide, bearing a five-year survival rate of less than 10% [23,24]. The peculiar nature of pancreatic cancer stems from its propensity for early metastasis and resistance to conventional treatment [25,26,27]. Among the various manifestations of metastatic disease, cutaneous metastasis emerges as a relatively uncommon but clinically significant marker of systemic progression and disseminated malignancy. Although cutaneous metastases from pancreatic cancer account for only 1–3% of all cutaneous metastases, their presence cannot be underestimated [15]. In this study, we analyzed the clinical features of skin metastases resulting from pancreatic cancer. Our analysis involved a cohort of 57 patients from different publications, allowing us to investigate the epidemiology, clinical presentation, and histological features of this rare manifestation.

### 4.2. Epidemiology and Clinical Presentation (Table S1)

Our cohort showed an almost equal distribution of skin metastases between males and females with a mean age of 63.4 years. The prevalence of skin metastases in pancreatic cancer appears to be relatively low, which is consistent with the rarity of this phenomenon [23,28]. Skin metastases were predominantly non-umbilical, occurring in 77% of cases, while umbilical metastases were less common, accounting for 23% of cases. Among the non-umbilical metastases, we observed that metastases of the head-neck district represented the most frequent skin metastases considering the single district (33%), followed by the trunk (21%). This distribution suggests that skin metastases from pancreatic carcinoma may occur in various anatomical regions, which may complicate early diagnosis. These data contrast with the systematic review by Gu et al. of 2023, entitled ‘Pancreatic cancer: Cutaneous metastases, clinical descriptors, and outcomes’, where the frequency of umbilical metastases was considered in relation to all other skin metastasis sites without grouping them into a group defined as ‘non-umbilical’ [15]. This distinction in categorization methodology highlights the importance of standardized definitions and classifications in clinical research to ensure consistency and comparability of results between studies. Clinically, cutaneous metastases of pancreatic cancer can present with a variety of skin lesions that are important for healthcare professionals to recognize. They may present as violaceous nodules, masses, and indurated plaques (89%) with a significant proportion of asymptomatic lesions (34%). Lesions may be centrally erosive or associated with soft tissue prominence. Other presentations include brown verrucous, erythematous plaques, or lesions with a purplish raised border and ulcerated center. The nodules may be fixed, non-inflamed, or associated with intermittent foul-smelling discharge, suggesting infection. In some cases, there may be rapid expansion of the oedema, suggesting a rapidly growing tumor. Cutaneous ulceration is also common and may present with smaller ulcers surrounding a larger lesion, often with multiple subcutaneous nodules. An infiltrative area of coalescing ulcerated papules, particularly around the umbilicus, is another telltale sign. The nodules may vary from well-circumscribed, smooth, erythematous, and firm to lesions with central ulceration and crusting. In addition, erythematous macules and nodules may surround these ulcerated areas. Lesions may also have a blackish tinge, be tender, and be attached to the skin with a serous discharge, indicating an underlying infectious process or necrosis [29,30,31]. These findings highlight the different possible clinical presentations of skin metastases in pancreatic cancer, which may mimic other dermatological conditions, making early recognition difficult [11,17].

### 4.3. Characteristics of Pancreatic Cancer

The primary site of pancreatic cancer was predominantly in the body or tail (44%) and head (42%). The size of the primary tumor was more frequently less than 4 cm (52%), indicating that skin metastases may arise from relatively small pancreatic tumors. Adenocarcinoma was the most common histological type associated with skin metastases (77%), followed by neuroendocrine tumors (7%) and sarcoma (4%). At the time of diagnosis of skin metastases, patients often presented with gastrointestinal symptoms (44%), jaundice, and weight loss (both 20%). Lymph node metastases were detected in 45% of cases, while distant metastases were more widespread, particularly in the liver (54%) and lungs (31%). This suggests that cutaneous metastases in pancreatic cancer often occur in the context of advanced disease.

### 4.4. Correlation between the Timing of Presentation of Skin Metastases and the Diagnosis of Pancreatic Cancer (Figure 3)

In our study, we conducted a Pearson’s Chi-square test to explore the potential relationship between the timing of presentation of skin metastases and pancreatic cancer diagnosis. The results revealed a statistically significant association (*p*-value = 0.008415) between these two variables. Specifically, we observed that pancreatic tumors located in the body and tail of the pancreas were more frequently associated with skin metastases as the first manifestation of the tumor (62.2%) than pancreatic tumors located in the head of the pancreas (20.8%). This finding suggests that patients with pancreatic tumors in the body and tail of the pancreas are more likely to develop skin metastases as the first clinical manifestation [32,33]. A possible explanation for this observation is that tumors in the body and tail of the pancreas tend to remain clinically silent for a longer period compared to those in the head of the pancreas. In fact, pancreatic head cancers often cause early symptoms due to their proximity to the bile duct and duodenum, resulting in jaundice, weight loss, and other gastrointestinal disorders [34,35,36]. In contrast, lesions in the body and tail can grow silently until they reach a more advanced stage, when they are more likely to metastasize to distant locations, including the skin [34,35]. 

## 5. Limitations

It is important to recognize that our study has some limitations, including the retrospective nature of data collection and the relatively small sample size. In addition, we only included pancreatic cancer cases in which skin metastases were reported and described. However, we do not know the true prevalence and incidence of these disease manifestations, as they are not always reported or researched. Larger scale studies would be needed to further validate our findings and provide a more complete understanding of the relationship between pancreatic cancer location and the occurrence of skin metastases. However, we stress the clinical importance of recognizing potential differences in metastatic behavior depending on the location of the pancreatic primary and emphasize the importance of vigilant monitoring and early diagnosis in these patients.

## 6. Conclusions

This comprehensive study explored the relationship between pancreatic cancer and skin metastatic spread. Despite its rarity, skin metastases could have some implications for providing the best possible patient care through earlier diagnosis and management of the disease. The prevalence of non-umbilical skin metastases, especially in the head and neck region, requires physicians to maintain a high index of suspicion, as these lesions may mimic other dermatological conditions. The clinical presentation is heterogeneous, ranging from asymptomatic nodules to painful or ulcerated lesions, making their early recognition a clinical challenge. The prevalence of primary pancreatic tumors in the body and tail as precursors of skin metastases confirms the insidiousness of distal pancreatic cancers, which often remain clinically silent until they reach an advanced stage. In such cases, skin metastases can represent the first clinical manifestation, unlike primary tumors located in the head of the pancreas, which tend to manifest earlier due to their proximity to critical anatomical structures. In conclusion, this study reinforces the importance of early recognition of cutaneous metastasis and interdisciplinary collaboration in the management of pancreatic cancer. Skin metastases, although rare, represent a visible warning sign that should not be overlooked in clinical practice. 

## Data Availability

The data presented in this study are available on request from the corresponding author.

## Supplementary Materials

The following supporting information can be downloaded at: https://www.mdpi.com/article/10.3390/jcm13010104/s1, Table S1: Cutaneous metastases presentation.
