# Supplementary material for: The Importance of Reading the Skin: Cutaneous Metastases of Pancreatic Cancer, a Systematic Review
Source: J Clin Med. 2023 Dec 24;13(1):104. doi: 10.3390/jcm13010104 (PMC10779471; doi:10.3390/jcm13010104)
Supplement: Supplementary file 1 [file jcm-13-00104-s001.zip › jcm-2753892-supplementary.pdf]

Journal of
Clinical Medicine

MDPI

Supplementary Table S1. Cutaneous metastases presentation

Authors	Year	Clinical presentation	Authors	Year	Clinical presentation
Abdel Hafez	2008	Violaceous nodules and indurated plaques	Miyahara et al.	1996	Firm painless nodule
Abdelaziz et al.	2022	Node with central erosive changes	Miyahara et al.	1996	Firm painless nodule
Aghighi	2021	Soft tissue prominence	Miyahara et al.	1996	Firm nodule with a purulent secretion
Ambro et al.	2006	Brown verrucous erythematous indurated plaque	Miyahara et al.	1996	Three firm painful nodules
Arya	2012	Painful and itchy mass	Miyahara et al.	1996	Hard cutaneous induration
Atmatzidis et al.	2022	Palpable hard and painful mass	Moon JI et al	2016	Firm, painful, and non-pruritic, with a central plug nodule
Aydin et al.	2005	Painless swelling	Nasser	2021	T2 dermatomal rash with hyperpigmented indurated plaque with few satellite papules
Bai et al.	2012	Swelling, redness and induration	Nawashiro	2002	Subcutaneous painless mass
Bdeiri K et al	2013	Painful lesion	Pandey P et al	2016	Slow growing lesion
Bhardwaj	2017	Redness and swelling with intermittent malodorous discharge	Pontinen	2010	Not clinically informative
Boysen	2011	Several erythematous elevated plaques	Prabhu	2013	Blackish nodule
Chakraborty	1977	Nodular, tender, bluishpink mass and outline. Firm, irregular node	Ram	2006	Yellow nodule
Chapman	1989	Firm nodule	Ramachandran P et al	2020	Asymptomatic lump
Fagan	2018	Painful cutaneous nodules	Rashleigh-Belcher HJ et al	1986	Cutaneous nodule
Flórez A et al	2000	Round, indurated mass, with an ulcerated centre	Reyes García-De La Fuente M et al	2013	Cutaneous nodules
Gawrieh et al.	2002	painless non pruritic papule in right temporal region	Saif MW et al	2011	Discoloration and thickening of the skin
Green et al.	1952	Reddened plaque	Salari	2020	Violaceous nodule
Hisham Zayan et al	2008	Violaceous nodules, indurated plaques	Shi	2020	Red swollen papules
Horino et al.	1999	Firm, fixed, painless, noninflamed nodule	Shin	2015	Mass
Ito H et al	2020	Ulcer with exudate	Siriwardena et al.	1993	Swelling
Jun DW et al	2005	Multiple subcutaneous nodules	Sironi et al.	1991	Firm nodular mass
Kaoutzanis C et al	2013	Reddish, tender, firm nodule	Solin et al.	1983	Subcutaneous nodule
Kim et al.	2018	Firm, erythematous, round, tender nodule	St Peter et al.	2003	Subcutaneous nodule
Kotsantis I et al	2016	Bilateral oedema of the lower limbs	Takemura et al.	2007	Well-circumscribed, round, smoothsurfaced, erythematous, firm nodule
Kuśnierz et al.	2021	Rapidly enlarging tumour	Taniguchi	1994	Well-circumscribed, slightly indurated erythematous plaques with central ulceration and crusting. Several erythematous macules and nodules
Leyrat B et al	2021	Indurated nodules	Tavío-Hernández	2015	Firm nodules, some with ulcerated surface covered with small clots
March-Rodriguez A et al	2023	Cutaneous ulceration with two smaller ulcers adjacent to the first ulcerated lesion and multiple subcutaneous nodules	Van Akkooi	2010	Two skin lesions supposed to be pyogenic granulomas
Masmoudi et al.	2008	Infiltrative area with coalescing ulcerated papules at the umbilicus	Yanofsky V. R. et al.	2019	Tender, erythematous to violaceous, deep-seated subcutaneous nodule
Zhou H. et al.	2014	Asymptomatic violaceous nodules	Yendluri V. et al.	2007	Indurated mass
